# Cervical and Breast Cancer: Uncontrolled Epithelial Growth Driven by Xenoestrogen Dominance, Disrupted Paracrine Signaling, and Arrested Apoptosis

**DOI:** 10.3390/cells15211956

**Published:** 2026-10-27

**Authors:** Albert Despot, Ivan Sabol, Zlatko Hrgović, Ana-Marija Despot, Ivica Vlatkovic, Mara Tešanović, Dora Fureš, Sanja Malinac Malojčić, Vesna Ćosić, Nikola Sinković, Rajko Fureš

**Affiliations:** 1 Clinic for Gynecology and Obstetrics, University Hospital Centre Zagreb, 10000 Zagreb, Croatia; 2 Division of Molecular Medicine, Ruđer Bošković Institute, 10000 Zagreb, Croatia; 3 Goethe University, 60629 Frankfurt am Main, Germany; 4 Faculty of Food Technology and Biotechnology, University of Zagreb, 10000 Zagreb, Croatia; 5 Gynecology Practice Dr Vlatković, 23000 Zadar, Croatia; 6 Department of Obstetrics and Gynecology, Dubrovnik General Hospital, 20000 Dubrovnik, Croatia; 7 Department of Neurology, Zabok General Hospital and Croatian Veterans Hospital, 49210 Zabok, Croatia; 8 Department of Obstetrics and Gynecology, Zabok General Hospital and Croatian Veterans Hospital, 49210 Zabok, Croatiarajko.fures@bolnica-zabok.hr (R.F.); 9 Faculty of Dental Medicine and Health, Josip Juraj Strossmayer University of Osijek, 31000 Osijek, Croatia; 10 Polyclinic Cosic, 35000 Slavonski Brod, Croatia; 11 Croatian Society of Pelviperineology, 49210 Zabok, Croatia; 12 Faculty of Health Studies, University of Rijeka, 51000 Rijeka, Croatia

**Keywords:** breast cancer, cervical cancer, xenoestrogens, apoptosis, paracrine signaling

## Abstract

Cervical cancer and breast cancer represent two serious challenges in modern gynecological oncology. Despite their anatomical accessibility for screening, significant investments in research, and the development of therapeutic strategies, their global incidence and mortality rates continue to rise. Interstitial spaces, paracrine signaling, hormonal imbalance linked to xenoestrogens (XE) and the consequent inhibition of apoptosis constitute a common, yet unfortunately insufficiently recognized, link in the carcinogenesis of both cervical and breast cancer. The aim of this paper is to supplement the classical paradigm of cervical and breast carcinogenesis by incorporating a broader context currently missing from the understanding of both forms of cancer. XEs are widely distributed chemical substances, found in a wide range of everyday products, food and environment, that mimic the action of endogenous estrogen. In the world of basic science, the connection between estrogen dominance and apoptosis avoidance is unquestionable and can undeniably impact progression of breast and cervical cancer. In the development of breast cancer, the association between XE facilitated estrogen dominance and arrested apoptosis is of great importance. In the pathogenesis of cervical cancer, the activity of viral oncogenes E6/E7 is the main driver of the pathological condition; however, in the context of arrested apoptosis, there is an area of overlap between estrogen dominance and HPV virus action. In this sense, estrogen dominance, triggered by the agonistic action of XEs on the membrane-bound GPER receptor of fibroblasts, is a cofactor in cervical carcinogenesis that remains insufficiently researched.

## 1. Introduction

Breast cancer is the most common cancer affecting women worldwide and leading cause of with forecasts suggesting further increases in incidence and mortality worldwide [1]. Without further interventions, many countries will fall short of WHO Global Breast cancer Initiative [2]. Similarly, cervical cancer remains the most common gynecological malignancy in women targeted by another dedicated WHO elimination initiative [3]. Concerningly both breast and cervical cancer burden rose worldwide and in all but the highest developed regions [4]. Both cancer types are also attributable to modifiable risk factors [5] and both appear to interact with estrogen and endocrine disruptors [6,7,8].

Estrogens are crucial for sustaining life, reproduction, and a woman’s physiological health. A decline in estrogen levels or activity affects more than just the reproductive capacity of the human species. Estrogens are essential for the functioning of the skeletal, cardiovascular, and nervous systems, and they play a significant role in skin and adipose tissue homeostasis [9]. Excessive estrogenic activity or the mimicry of estrogenic activity, as is the case with xenoestrogens (XEs), can cause functional disorders or even the development of cancer in certain estrogen-sensitive organs [10,11].

Classic estrogen signaling predominantly relies on estrogen receptor (ER) alpha (ERα), localized within the nuclei of epithelial and glandular cells of the uterus, ovaries, and breasts [12]. Through these conventional ER-mediated mechanisms, endogenous estrogens actively promote cellular proliferation while inhibiting apoptosis [13]. Apoptosis, the genetically programmed execution of cell death, serves as a critical defense mechanism required for tissue regeneration [14], the clearance of genomically damaged cells [15], but also possible roles in cancer prevention [16].

Xenoestrogens represent a ubiquitous class of environmental substances that can interfere with the physiological functioning of the endocrine system by mimicking the action of endogenous estrogens, such as estradiol (E2) [17]. The most well-known sources of XEs are plants, pesticides, plastics, cosmetic products with new sources of XE in the environment increasing through new industrial and agricultural advancements [11]. Beyond interacting with ERs, XE also interact with G-protein-coupled estrogen receptors (GPERs, formerly GPR30) [18], which are prominently expressed on not only the membranes of interstitial fibroblasts [19] but also other compartments within reproductive and mammary tissues [20]. Under pathological conditions, the aberrant activation of GPER by environmental XEs triggers alternative non-genomic signaling cascade that can contribute to a state of arrested apoptosis, ultimately disrupting tissue homeostasis and driving cervical and breast oncogenesis [20].

However, interstitial signaling dynamics remained underappreciated and unexplored, primarily due to limitations of traditional histological techniques. Only after 2018, when researchers using probe-based confocal laser endomicroscopy (pCLE) to visualize living tissue was a vast, macroscopic interstitial network identified that had previously evaded clinical detection [21]. Benias et al. observed in vivo an interstitial structure that almost completely disappears during the preparation of samples for microscopic analysis. The chemical dehydration required for tissue processing extracts interstitial fluid and forces the structural collagen network to collapse. As a result, this highly active paracrine signaling corridor was rendered physically invisible under conventional microscopy and remained a terra incognita for pathologists and oncologists alike. More concerningly, these discoveries have not entered contemporary clinical consideration despite the discovery being fairly old.

Recent frameworks of cervical carcinogenesis identified arrested apoptosis as a primary generator of malignant transformation, allowing human papillomavirus (HPV) to evade programmed cell death and drive CIN progression [22]. However, the paradigm shifting discovery of the interstitial spaces as a dynamic conduit for paracrine communication highlights the need to revisit our oncogenic models. To address this, we have queried PubMed, Web of Science and Scopus databases for studies focusing on estrogen signaling and specifically paracrine and non-genomic version of estrogen signaling as well as XEs in breast and cervical cancer contexts. The relevant manuscripts and references therein were screened for further relevant publications. The review was not intended as exhaustive or systematic.

Ultimately, the goal of this narrative review is to bridge the critical gaps in previous oncogenic models by integrating viral factors and arrested apoptosis with the dynamics of interstitial paracrine signaling and XE-driven estrogen dominance [23,24]. By uniting mechanisms that have historically been either neglected or evaluated in isolation, this manuscript provides a comprehensive, microenvironment-driven framework for understanding and thinking about both cervical and breast carcinogenesis to support further efforts of breast and cervical cancer control.

## 2. Apoptosis and Estrogen Influence Tissue Dynamics

Apoptosis is a highly regulated mechanism of programmed cell death essential for maintaining tissue homeostasis, eliminating genomically damaged cells, and preventing the propagation of viral infections and cancer [14,25,26]. While the apoptosis pathway is activated by both intracellular (intrinsic–mitochondrial) and extracellular signals (extrinsic–death receptor pathway), in the context of epithelial renewal, the intrinsic pathway is the primary arbiter of cell fate [27]. This pathway is governed by the Bcl-2 protein family, which acts as the critical sensor and guardian of mitochondrial integrity [28] containing both pro and anti-apoptotic members. Briefly cellular stress like DNA damage or oxidative stress activate pro-apoptotic proteins, including multi-domain executioners (BAX, BAK) and BH3-only sensitizers (BAD, PUMA, NOXA) [28]. These proteins promote mitochondrial outer membrane permeabilization, triggering the release of cytochrome c into the cytoplasm [29,30].

Upon translocation, cytochrome c binds to APAF-1 and caspase-9 to form the apoptosome, a multiprotein complex that ignites a proteolytic cascade [31,32,33]. This activates executioner caspases (caspase-3 and -7) that systematically cleave nuclear DNA and dismantle the cell [34,35]. Conversely, anti-apoptotic proteins residing in the outer mitochondrial membrane, specifically Bcl-2 and Bcl-xL, bind to and neutralize these pro-apoptotic factors to ensure cell survival [36,37]. The dynamic, competitive balance between these pro- and anti-apoptotic forces represents a critical regulatory node, one that is highly vulnerable to disruption by aberrant extracellular survival signals. However, non-physiological perturbations can block the programmed cell death pathway and by preventing apoptosis push the cells to senescence and if extensive exacerbate pathological states via senescent associated secretory phenotype (SASP) [38,39]. In cervical cancer, for example, HPV is undeniably known to prevent apoptosis by interactions with p53 which we termed arrested apoptosis previously [22]. Furthermore, HPV and estrogen are also known to interact further complicating the issue [40,41].

During the reproductive years, the epithelial tissues of the cervix [7], endometrium [42], ovaries [43], and breast [44] undergo intense proliferative cycles driven by estrogenic stimuli. Endogenous estrogens, primarily E2, act as potent pleiotropic regulators necessary for maintaining numerous reproductive and non-reproductive physiological functions [6]. The biosynthesis of endogenous estrogens involves the conversion of androgens (androstenedione or testosterone) through the process of aromatization, yielding estrone (E1), 17β-estradiol (E2), estriol (E3), and estetrol (E4), which primarily occurs in the ovaries but also in other tissues [45,46].

By binding to classical nuclear ERs (ERα and ERβ), E2 can activate transcription of growth factors and ultimately elicit effects on proliferation, apoptosis, differentiation and immunity [47,48]. In addition to classic nuclear ERs, estrogens engage cell surface receptors, specifically GPER, to trigger rapid, non-genomic cellular responses via rapid intracellular kinase cascades [49].

Concurrently to promoting proliferation, within physiological limits, estrogenic activity provides short-term survival signals by inhibiting pro-apoptotic proteins (BAK, PUMA) and upregulating the anti-apoptotic activity of Bcl-2 and Bcl-xL [13,50]. Physiologically, this proliferative burst is strictly counterbalanced by progesterone during the luteal phase [51], which actively reinstates apoptotic clearance of damaged or dysfunctional cells generated during rapid mitosis [52,53].

Although estrogens are crucial for normal tissue development and the menstrual cycle, increased exposure significantly increases the risk of cellular hyperplasia and neoplastic transformation in sensitive tissues [7,54,55,56] by establishing the pathological state called estrogen dominance [57]. While clinical estrogen dominance is classically viewed as a systemic progesterone deficiency driven by aging, lifestyle, or exogenous hormone therapies, localized paracrine XE-driven estrogen dominance is of rising concern.

## 3. Physiological and Pathological Dynamics of the Cervical Epithelium: Bridging the Gap in Paracrine Signaling

During the reproductive period, the ectocervical epithelium undergoes continuous remodeling through coordinated proliferation, maturation, and desquamation, achieving complete cellular turnover every 4–5 days [58]. ERs are primarily localized within the nuclei of the basal, parabasal, and intermediate cell layers [59,60]; accordingly, the administration of estrogenic compounds can rapidly accelerate the maturation of the squamous epithelium to three days [61]. Unlike the endometrium, the endocervix exhibits only marginal fluctuations in ER expression between the follicular and luteal phases [62], while atrophic or severely inflamed exocervical epithelia demonstrate significant receptor depletion. Progesterone receptors are largely absent in the ectocervical epithelium during the follicular phase but become detectable in the parabasal layer during the luteal phase and pregnancy [59]. Crucially, however, histological data confirms that both estrogen and progesterone receptors are persistently expressed at high densities within the underlying ectocervical stromal fibroblasts throughout the entirety of the menstrual cycle [58]. Because these interstitial fibroblasts exhibit a markedly higher receptor density than the overlying epithelium, the conventional model of direct, endocrine-driven epithelial proliferation is mechanistically incomplete, and must be revised to account for stroma-driven, paracrine-mediated regulation.

Due to the sparse vascularization of the cervical surface epithelium, which lacks the basal or superficial arterial networks found in the endometrium [63], endocrine signals originating from distant organs cannot easily orchestrate rapid metabolic or proliferative responses during periods of intense proliferation. Conversely, the underlying stromal interstitium is permeated by a dense capillary network [64]. To compensate for epithelial avascularity, tissue homeostasis in tissues undergoing rapid epithelial renewal likely shifted towards relying stromal–epithelial paracrine signaling triggered by endogenous estrogens, particularly estradiol (E2) [65].

Estrogens can trigger the classic genomic pathway by binding ER in the cytoplasm which after a conformation change translocates to the nucleus and acts as transcription factor affecting gene transcription and leading to diverse physiological outcomes. In addition, it has been shown that estrogen, along with other steroid hormones, can also elicit strong non-genomic signaling through membrane bound ER and GPER receptors [65,66,67], which is often neglected and thus worth emphasizing:

- A classic hormone response genomic pathway (operating over hours to days) via gene transcription.
- A rapid non-genomic pathway (operating within seconds to minutes) driven by kinase cascades or calcium flux, and affecting diverse signaling pathways including MAPK/ERK, and PI3K/Akt signaling.

**Figure 1:**
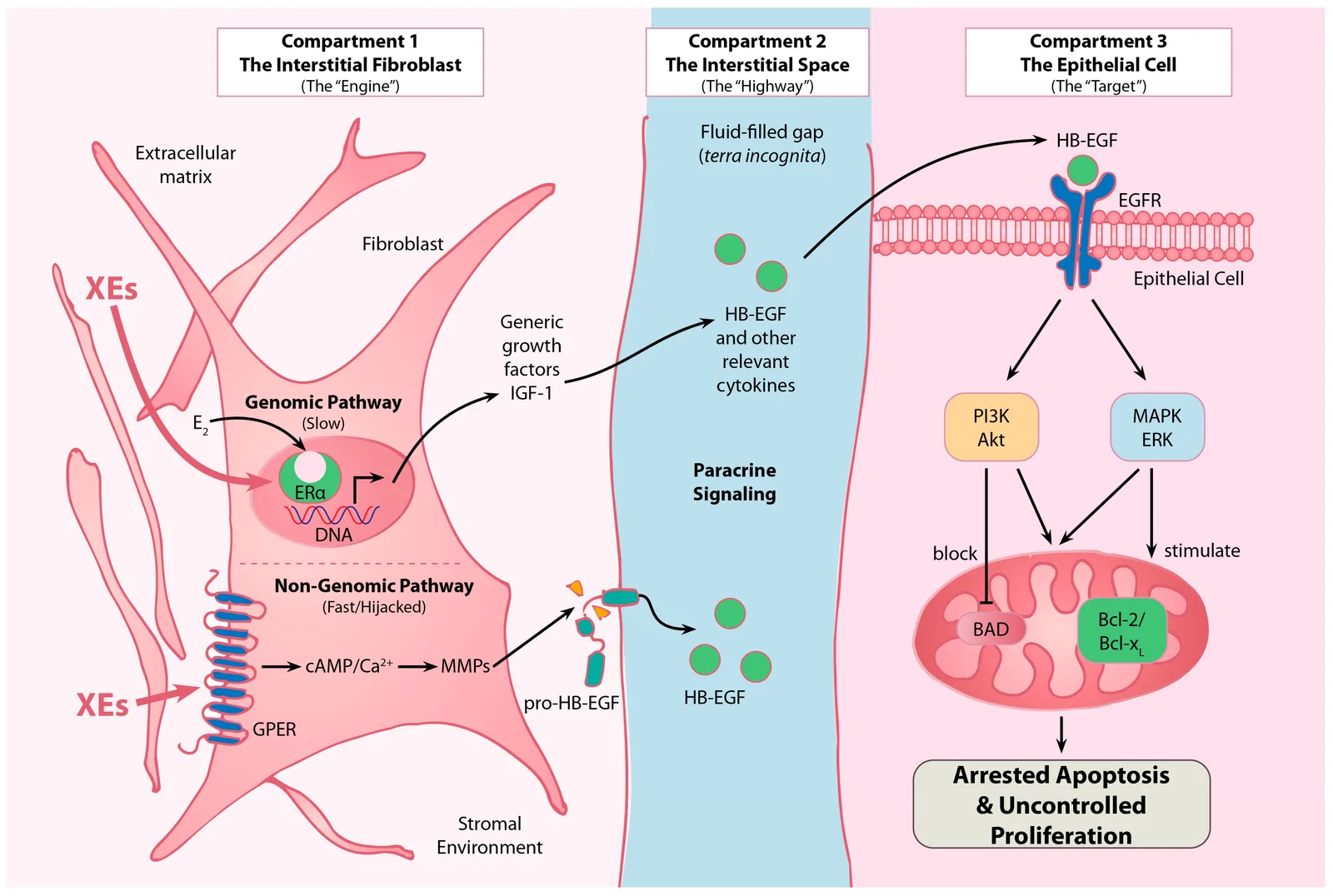
Classic genomic and non-genomic pathways activated by estrogen.

Rather than relying on nuclear transcription factors, the rapid non-genomic pathway found in the paracrine system operating via rapid signaling cascades and messenger systems is activated by estrogen (E2) binding to GPER found on fibroblasts [20,68] (Figure 1). Initial E2 binding leads to increased production of cAMP and mobilization of intracellular calcium (Ca^2+^) [49]. Simultaneously, there is rapid activation and release of matrix metalloproteinases (MMPs), which cleave the membrane-bound precursor of heparin-binding EGF-like growth factor (pro-HB-EGF) and release its soluble form [69]. Subsequently, the released HB-EGF immediately transactivates the EGF receptor (EGFR) on the same fibroblast, triggering an autocrine signaling loop that rapidly activates the downstream MAPK/ERK and PI3K/Akt signaling pathways [70,71]. Both pathways are also known to interact with Bcl-2 family of proteins, i.e., blocking activity of Bad via phosphorylation and upregulating Bcl-xL levels among others, ultimately have an antiapoptotic effects [50]. This robust fibroblast activation culminates in the interstitial secretion of pro-proliferative, anti-apoptotic but also pro inflammatory mediators and cytokines, which then act via paracrine diffusion to accelerate epithelial proliferation.

While this intricate stromal–epithelial cross-talk is essential for healthy cervical turnover, GPER non-genomic estrogenic signaling has been implicated both in cervical, breast and other gynecological cancers [20]. The environmental XEs further disrupt this balance by infiltrating these poorly vascularized interstitial spaces. Prolonged XE overstimulation may drive fibroblasts into a state of chronic, aberrant paracrine secretion, subverting physiological renewal and establishing biochemical conditions for arrested apoptosis and oncogenesis in estrogen-sensitive tissues.

## 4. Xenoestrogens in the Pathogenesis of Breast and Cervical Cancers

Xenoestrogens constitute a diverse group of exogenous compounds, belonging to the broader class of endocrine-disrupting chemicals (EDCs), that are uniquely capable of mimicking or antagonizing endogenous estrogen signaling [72]. It has been long known that external estrogen can affect cancer development [73]; however, the hypothesis linking environmental estrogenic disruptors to carcinogenesis gained significant traction in the early 1990s [74], driven by epidemiological studies investigating organochlorine pesticides (e.g., DDT and its metabolite DDE) and polychlorinated biphenyls (PCBs) in breast cancer cohorts [75].

While early reports suggested a strong correlation, subsequent large-scale analyses yielded conflicting results [76,77], sparking controversies that ultimately refined modern endocrine disruption research. Today, XEs are broadly categorized into phyto/mycoestrogens (plant or fungi derived), metalloestrogens, and synthetic industrial, agriculture or household chemicals, which has recently been thoroughly reviewed [11]. The XEs are known to elicit effects on both pertinent cancer types, breast [78], cervical [79] as well as combined gynecologic cancers [8], and each of these associations were comprehensively reviewed. Thus, we present herein only the brief overview of the fields relevant for understanding the wider concepts.

**Figure 2:**
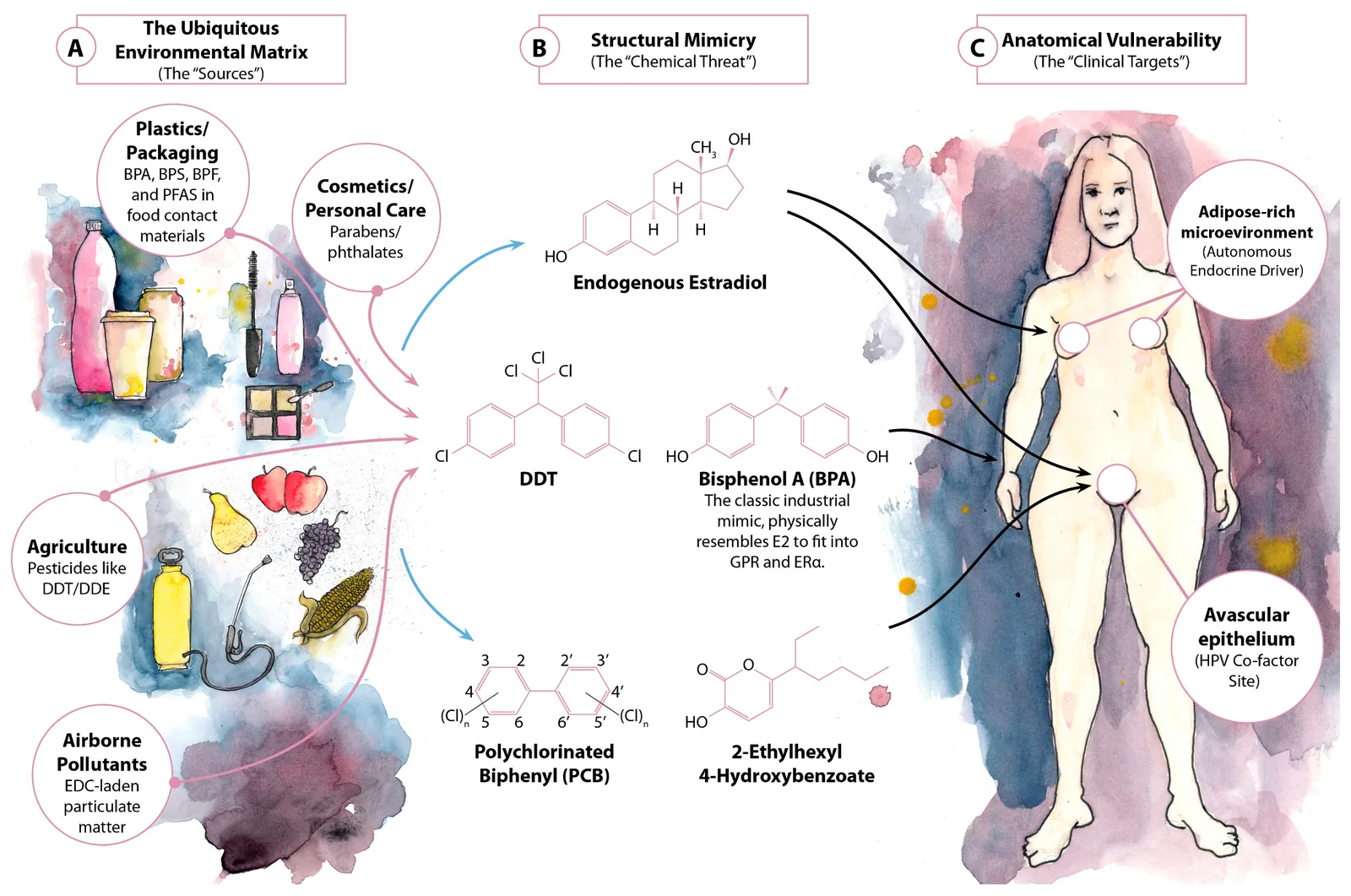
Sources of xenoestrogen exposure (**A**), structural mimicry (**B**) and local contexts in gynecologic cancer development (**C**).

Modern exposure stems (Figure 2) from a ubiquitous environmental matrix including synthetic industrial applications, plastics [80], agricultural contaminants [81,82] or food [83], personal care products and cosmetics [84], as well as airborne pollutants [85] carrying endocrine-disrupting particulate matter.

The inescapable nature of this exposure has recently forced unprecedented legislative action, highlighting the urgency of this clinical challenge. Within plastics manufacturing, regulatory crackdowns on bisphenol A (BPA) merely led to its substitution with structurally similar analogues (e.g., BPS and BPF), which demonstrate comparable or even heightened estrogenic and oncogenic potential [86]. Concurrently, per- and polyfluoroalkyl substances (PFAS) now dominate toxicological and regulatory attention due to their extreme environmental persistence. In the European Union, a comprehensive REACH restriction proposal covering over 10,000 PFAS compounds [87,88] is advancing, alongside recent measures like the August 2026 ban on PFAS in food contact packaging under the Packaging and Packaging Waste Regulation. While in vitro assessments of isolated PFAS binding to classical ERs have occasionally yielded conflicting results [89,90,91], their role as potent endocrine disruptors is undeniable. When evaluated in real-life serum and placental mixtures, specific PFAS compounds and persistent pollutants consistently exhibit profound XE activity and correlate directly with endocrine disruption in women [92,93,94,95,96,97].

A primary methodological hurdle in assessing XE-driven oncogenesis is the phenomenon of mixture toxicity. Endocrine disruptors frequently defy classical linear toxicology by exhibiting non-monotonic dose–response (NMDR) curves, where ultra-low environmental doses provoke stronger receptor-activating effects than higher concentrations [98]. This is compounded by continuous human exposure to low-dose mixtures from different sources [99]. However, combined low dose airborne pollutant XE mixture was shown to affect breast cancer risk [100]. Toxicological models repeatedly demonstrate that additive combinations of different XEs easily bypass single-chemical safety thresholds [101,102,103]. Evaluating these mixtures is uniquely difficult because individual XEs frequently exhibit dualistic or selective behavior, either functioning as estrogen agonists in certain cellular contexts or antagonists in others [104]. However, when evaluating the total effective XE burden in breast cancer patient serum, significant associations were found [105]. Furthermore, it is often difficult to examine exposure times and susceptibility periods [17,106], leading to inconsistencies in cancer associations even for the most often studied compounds like Bisphenol A in breast cancer [77] or cervical cancer [107] despite its biological effects [108].

The impact of XEs on cervical cancer represents an under-recognized but clinically relevant mechanism. While cervical carcinogenesis is fundamentally driven by persistent high-risk human papillomavirus (HPV) infection, estrogen signaling can likely act as a co-factor [109]. Exogenous estrogenic stimulation was shown to actively enhance the transcription of viral oncogenes E6 and E7 [40,110]. Consequently, elevated estrogen levels accelerate tumor growth in in vivo mouse models [111], whereas blocking ER signaling suppresses cervical cancer cell proliferation [112,113]. Sustained exposure to environmental XEs therefore acts as a persistent environmental promoter in HPV-infected tissues.

Ultimately, at the cellular level, XEs exert these oncogenic effects not just through classical nuclear ERα and ERβ, but through dynamic cross-talk with membrane-associated receptors [17]. As detailed in the following sections, it is the continuous, XE-driven perturbations of these non-genomic paracrine signaling pathways within the interstitial microenvironment that paralyzes normal cellular defenses, establishing the estrogen-dominated conditions required for unchecked epithelial growth and the evasion of apoptosis.

## 5. Paracrine Non-Genomic Signaling and Xenoestrogens: A Hidden Driver of Uncontrolled Epithelial Growth

In contrast to the tightly regulated signaling of endogenous estrogens, XEs subvert this physiological pathway by binding to GPER and mimicking E2 in an unchecked and potentially continuous manner [114] (Figure 1). This can induce a state of localized estrogen dominance, effectively dismantling physiological feedback loops [115] as well as locking the downstream paracrine signaling cascade into a constitutively active state [20,49]. This persistent activation and altered cellular microenvironment, including unregulated expression of the HPV E6 and E7 oncogenes can lead to arrested apoptosis and cancer onset [109,111]. From an evolutionary standpoint, biological systems fundamentally rely on the dualistic principles of homeostasis and regulated cell death. However, paracrine control of cervical epithelial proliferation remains highly vulnerable to non-physiological disruption. Over the last century, the proliferation of synthetic chemicals has been proposed to contribute to a hazardous pathogenic triad within human tissues: environmental XEs, persistent local estrogen dominance, and arrested apoptosis. This combination may disrupt the normal growth remodeling of the epithelium, ultimately facilitating cancer development.

## 6. Xenoestrogen-Mediated Paracrine Signaling: Evasion of Apoptosis in the Estrogen-Dominated Breast Tumor Microenvironment

The mammary gland microenvironment represents a highly dynamic niche where complex epithelial–stromal interactions dictate cell fate. Within this architecture, local adipose tissue serves not merely as structural support, but as a highly active endocrine organ capable of maintaining an estrogen-dominant milieu through autonomous peripheral androgen to estrogen conversion [116]. Emerging evidence underscores that this delicate paracrine network is exceptionally vulnerable to disruption by XEs [10,11]. By hijacking membrane-bound receptors such as GPER alongside classical ER pathways, these endocrine-disrupting chemicals bypass physiological feedback mechanisms [117] culminating in arrested apoptosis and driving uncontrolled proliferation in breast cancer pathogenesis.

The cellular composition of the breast microenvironment actively facilitates this disruption. Adipose tissue progenitor cells, particularly those expressing PDGFRα, exhibit significant plasticity under the influence of environmental and oncogenic signals [118]. Contact with malignant cells stimulates these progenitors to differentiate into cancer-associated fibroblasts (CAFs), which aggressively secrete pro-inflammatory cytokines that facilitate tumor expansion and therapeutic resistance [119]. Concurrently, mature adipocytes act as a massive reservoir for paracrine and endocrine factors [120]. Since the discovery of leptin, over 400 adipocyte-derived factors (adipokines) across different tissues have been identified, including growth factors, chemokines, and adiponectin [121,122,123]. Investigating the role of adipocytes in the development or progression of cancer is of great clinical interest due to the established connection between obesity and cancer [124]. Cancer-associated adipocytes (CAAs) exhibit increased secretion of pro-inflammatory cytokines such as IL-6 and TNF [125]. These cytokines act in a paracrine manner on adipocytes and stromal tissue, drastically upregulating aromatase (CYP19A1) expression [126]. This creates a vicious cycle of local hyperestrogenemia, independent of circulating estrogen levels. The presence of XEs in the sensitive microenvironment can further drive disease progression via GPER-mediated signaling. While classical ERα acts via the genomic pathway very slowly, XE can via GPER trigger immediate non-genomic pathway and activate paracrine signaling cascades leading to rapid activation of MAPK/ERK and PI3K/Akt signaling pathways in neighboring cells. Constant stimulation of Akt and ERK pathways triggered by the action of XE leads to decreased activity of pro-apoptotic proteins [127]. At the same time, there is increased expression of anti-apoptotic proteins from the Bcl-2 family [128]. The ultimate result is that, despite the accumulation of genetically damaged or mutated cells in the hypoxic microenvironment, the metabolic suspension of programmed cell death, or arrested apoptosis, becomes a reality, or more precisely, their fate.

## 7. Discussion

The mechanisms elucidated in this article shift the paradigm of hormone-driven carcinogenesis from genome-centric model to one fundamentally driven by the microenvironment through paracrine non-genomic pathways. While classical endocrinology often considers estrogenic responses across tissues to be uniform, this article underscores the importance of distinguishing between viral and non-viral mechanisms of oncogenesis.

In cervical cancer, for example, estrogen pathways mediated by stromal paracrine signaling act primarily as an obligate cofactor that inhibits programmed cell death. This microenvironmental hijacking supports the high-risk HPV oncoproteins E6 and E7, which otherwise directly degrade the tumor suppressors p53 and Rb.

Conversely, the development of malignancy in the mammary gland does not require a viral initiator; instead, the abundant fatty stroma acts as an autonomous endocrine driver. Within this niche, environmental XEs play an important role through local paracrine signaling via GPER. Activation of this non-genomic pathway promotes continuous, aberrant interactions between the epithelium and stroma, bypassing physiological feedback loops. The likely consequence of this XE-induced paracrine disorder is the molecular induction of arrested apoptosis.

By rendering mammary epithelial cells insensitive to programmed cell death, independently of any viral oncoproteins, this apoptotic blockade creates a self-sustaining and favorable niche. In this estrogen-dominated, microenvironmentally altered state, genomically damaged cells evade immune clearance, laying the foundation for the development of invasive breast cancer. Therefore, future oncological strategies and risk assessments should extend beyond the narrow consideration of systemic receptor profiles to focus on localized, paracrine factors that disrupt interstitial homeostasis through interactions with membrane-bound receptors.

## 8. Conclusions

This narrative review illustrates how the interplay between environmental xenoestrogens and suppressed apoptosis acts as a critical driver of hormone-dependent carcinogenesis across both non-viral (breast) and viral (cervical) etiologies. XEs represent ubiquitous endocrine disruptors whose cumulative impact on the interstitial microenvironment remains clinically underexplored. Because regulatory efforts frequently trigger the industrial adoption of similarly potent structural substitutes, human exposure and its associated oncogenic risks will likely present a growing concern. In current gynecological and oncological paradigms, the physiological burden of arrested apoptosis is frequently overlooked; yet, failing to eliminate genetically or virally damaged cells enables their survival and eventual transition into senescent or pre-malignant states that exacerbate disease progression. By combining insights into local estrogen dominance, GPER-mediated paracrine signaling, and apoptotic blockade within the interstitial niche, future research can pioneer targeted therapies, such as GPER antagonists or senolytics, to reactivate cell death and restore tissue homeostasis.

## Data Availability

No new data were created or analyzed in this study. Data sharing is not applicable to this article.

## Abbreviations

The following abbreviations are used in this manuscript:

XE	Xenoestrogen
ER	Estrogen receptor
GPER	G-protein-coupled estrogen receptor
E2	Estradiol
HPV	Human papillomavirus
CIN	Cervical intraepithelial neoplasia
PFAS	Per- and polyfluoroalkyl substances
EDC	Endocrine-disrupting chemicals
